# Dental Students’ Perceptions Towards E-learning in Comparison With Traditional Classroom Learning

**DOI:** 10.7759/cureus.51129

**Published:** 2023-12-26

**Authors:** Marwa Y Shaheen, Amani M Basudan, Abdulrahman M Almubarak, Abeer S Alzawawi, Fatemah M Al-Ahmari, Hajer A Aldulaijan, Hani Almoharib, Nahed Y Ashri

**Affiliations:** 1 Department of Periodontics and Community Dentistry, College of Dentistry, King Saud University, Riyadh, SAU

**Keywords:** online learning, face-to-face learning, e-learning, dental education, dental students, classroom learning

## Abstract

Introduction: Electronic learning (e-learning) has evolved into a popular educational approach since the coronavirus disease 2019 (COVID-19) pandemic. While this represents an additional model for teaching, traditional classroom learning fosters the development of interpersonal skills and enables students to share and discuss specific topics. However, existing research on the comparison of both these modes of learning in the field of dental education is inadequate. This study aimed to evaluate the perceptions of dental students towards both electronic and classroom learning.

Methods: A cross-sectional questionnaire-based survey was conducted between November 2022 and January 2023 among dental students in Saudi Arabia. Students were questioned on their comparative perceptions of e-learning and classroom learning before, during, and after the COVID-19 pandemic. Questionnaire responses, including demographic data, were collected and tabulated, using electronic data management software. The tabulated data were analyzed to provide descriptive statistics and compare electronic and classroom learning with demographic variables and previous experience with e-learning.

Results: Most respondents reported possessing average information technology (IT) skills and prior experience with e-learning. Blackboard Learning Management System (LMS) (Reston, VA: Blackboard Inc.), Zoom (San Jose, CA: Zoom Video Communications Inc.), and Microsoft Teams (Redmond, WA: Microsoft Corporation) were the most commonly used and advantageous e-learning platforms. While the majority of participants found both methods acceptable for problem-based learning sessions and theoretical lectures, they reported e-learning to be less effective than classroom learning for clinical and practical sessions. Regarding e-learning as a preferred method over classroom learning, most responses were "neutral" or "uncertain." Comparing the mean ranks of the ordinal responses for the different teaching methodologies and the nominal responses for e-learning as the preferred method, no statistically significant interactions were observed for demographic characteristics, IT-skill levels, or prior experience with e-learning.

Conclusion: Although enhanced performance and learning capacity are enabled through e-learning, the advantages of personal interactions and the feasibility of practical and clinical dental sessions are achieved only through classroom learning.

## Introduction

Electronic learning, or e-learning, is also referred to as web-based, online, distributed, computer-assisted, or Internet-based learning. It is a system of education wherein learners acquire information through the Internet and web-based applications. E-learning employs a pedagogical approach that demonstrates student-centered flexibility and facilitates student-faculty collaboration, despite the physical divide [1]. Over the last decade, e-learning has emerged as a popular teaching approach and represents a model for the effective delivery of education [2]. With the advent of high-speed internet and advanced web-based technologies, e-learning can utilize electronic tools and devices that ensure a smooth transformation from the traditional classroom learning model [2]. Because e-learning is based on remotely stored resources and lectures delivered from any location or as recordings, it enables greater accessibility and review of course content [3]. Furthermore, the digital nature of e-learning enables students to learn about the latest developments in their field of study apart from the knowledge acquired from their course content and classroom interactions [1].

A key attribute required for e-learning is technological literacy in operating computers and digital devices, which is further enhanced by prior exposure to e-learning through any one platform [4]. Although e-learning has been used in medical education for almost two decades, it has predominantly been employed as a teaching aid, which complements the knowledge and information acquired through face-to-face learning [5]. However, during the coronavirus disease 2019 (COVID-19) pandemic-induced lockdown and the ensuing physical distancing measures enforced worldwide, students and faculty members had no choice but to use e-learning as the only method of teaching [6]. Research has indicated that in this fast-paced and developing world, e-learning could emerge as a valuable teaching tool to continue education during crises, such as pandemics and wars [7]. While this was indeed beneficial during the COVID-19 lockdown, as educational programs in schools, colleges, and universities could continue without a break, challenges were encountered in the fields that require practical and psychomotor skill-based learning [8].

Personal interaction and frequent reinforcements are essential for any knowledge-sharing session aimed at cognitive and psychomotor skillset development [9]. Medical education, in general, and more specifically, dental education, is a learning system that emphasizes on didactic teaching and hands-on practical and clinical teaching. This includes practical sessions on laboratory models, problem-solving sessions based on clinical case-scenarios, and clinical sessions, wherein comprehensive dental treatment is provided by students under supervision [10]. Classroom learning facilitates the development of interpersonal relationships, enabling students to share and discuss their individual perceptions on a topic [11]. However, this traditional learning environment often lacks opportunities for repetitive learning and continued access to resources [12]. The aforementioned gap is largely filled by e-learning, wherein students are empowered to learn at their own pace and master the details of the study topic by reviewing course content, which is often recorded and stored online [11]. Moreover, the psychological well-being of students is challenged by e-learning and online education owing to isolation and a lack of student-faculty interaction [13].

Given the available evidence in the literature, it is interesting to note that both e-learning and classroom learning have their respective advantages and disadvantages. However, evidence comparing e-learning and classroom learning is unavailable for the area of dental education, which involves the development of competencies related to knowledge, cognition, and psychomotor skills. Particularly, considering scenarios, such as the recent COVID-19 pandemic lockdown, it is imperative to know more about student perceptions of e-learning as a methodology for dental education and its efficacy compared with traditional face-to-face classroom-based methodologies. Therefore, this study aimed to evaluate the perceptions of dental students about e-learning in comparison with traditional classroom-based learning.

## Materials and methods

The present study was designed as a cross-sectional survey from November 2022 to January 2023 to evaluate dental students’ perceptions of e-learning in comparison with traditional classroom-based learning in light of their experiences before, during, and after the “COVID-19 pandemic.” Dental university students in Saudi Arabia were the target population for this study and formed the sampling frame. The dental teaching curriculum in Saudi universities is spread over six years, with the first two years focusing mainly on didactic teaching. While simulated practical training on phantom models begins during the second year and progresses up to the fourth year, clinical training begins in the third year at a 50% level and increases gradually during the fourth and fifth years to reach 100% clinical training during internship [8]. Accordingly, students who had enrolled in their respective universities and completed at least one year of classes before the COVID-19 lockdown was imposed were included in the study. The following were excluded from the survey: the students who had dropped a course or semester during the lockdown because of technical or medical reasons and those who encountered the lockdown within a year of enrollment in the university. For the given study sampling frame, the sample size was estimated using “G*Power” open-source statistical software (version 3.1.9.7, 2020) (Düsseldorf, Germany: Heinrich Heine University Düsseldorf). Assuming an effect size of 0.3, alpha set at 0.05, and statistical power at 0.9, the desired sample size was estimated to be 240 participants [14].

Following an in-depth literature review, the research team prepared the survey questionnaire, altering it, based on the feedback received during the pilot phase, to ensure that the final survey questionnaire precisely collected the desired information from the target population. Survey questionnaires were distributed electronically, and delegated team members collected in-person data. This study aimed to evaluate the research question, both qualitatively and quantitatively.

Furthermore, the questionnaire consisted of four main parts as follows: (1) participants’ demographic information (age, gender, year of study in dental school), proficiency in IT skills (including but not limited to access and interaction using internet-based learning applications either through a personal computer or a handheld digital device), and prior experience with e-learning [11,15]. (2) Participants’ exposure to different e-learning platforms and their overall perception of the advantages of e-learning and classroom learning, with the ability to respond to multiple choices. (3) Students’ perceptions of e-learning and classroom learning for different teaching methodologies, including problem-based learning, theoretical lectures, clinical sessions, and practical sessions, wherein simulated clinical and practical sessions were acceptable. Participants ranked their responses on a four-point ordinal scale, namely, "e-learning is as good as classroom learning," "e-learning is better than classroom learning," "both learning methods are acceptable," and "e-learning is poorer than classroom learning." (4) Student responses on e-learning being preferable over classroom learning. Participants ranked their responses on a five-point nominal scale, namely, "strongly disagree," "disagree," "neutral/uncertain," "agree," and "strongly agree."

Data collection and statistical analysis

The data obtained were analyzed using the IBM SPSS (version 26.0 for Windows-based operating system) (Armonk, NY: IBM Corp.). Descriptive statistical analysis (mean, standard deviation, frequencies, and percentages) of the data obtained was conducted for the categorical and quantitative variables. The non-parametric Mann-Whitney U test was used to compare the mean ranks of the ordinal and nominal scale responses across the categorical study variables with two categories. For variables with three or more categories, the non-parametric Kruskal-Wallis test and the Conover test (for post-hoc analysis), were used to compare the mean ranks of ordinal and nominal scale responses across categorical study variables. For all statistical tests, the significance level was set at 95%, and any p-value less than 0.05 was considered statistically significant.

## Results

Descriptive analysis revealed that out of the 250 study subjects, 26 (10.4%) were younger than 21 years and 179 (71.6%) were in the age group of 22-25 years. With respect to gender distribution and nationality, 119 (47.6%) respondents were male and 241 (96.4%) were Saudi nationals. Based on the year of study at the dental school, 43 (17.2%) were studying in the third year, 74 (29.6%) in the fourth year, 57 (22.8%) were fifth-year students and the remaining 76 (30.4%) were interns and post-graduates. Most participants demonstrated an average level of IT skills (n=182, 72.8%) and reported prior participation and experience with e-learning activities (n=138, 55.2%). Descriptive statistics of the categorical variables are presented in Table 1.

**Table 1:** Participant demographics and their experience with e-learning (n=250). COVID-19: coronavirus disease 2019

Demographic characteristics and digital learning experience	n (%)
Age of the participants (years)	
<21	26 (10.4)
22-25	179 (71.6)
>26	45 (18.0)
Gender distribution	
Male	119 (47.6)
Female	131 (52.4)
Nationality of the participants	
Saudi	241 (96.4)
Non-Saudi	9 (3.6)
Level of study in dental school	
Third-year student	43 (17.2)
Fourth-year student	74 (29.6)
Fifth-year student	57 (22.8)
Intern	31 (12.4)
Postgraduate student	45 (18.0)
Level of information technology skills	
Above average	41 (16.4)
Average	182 (72.8)
Below average	27 (10.8)
Prior e-learning experience before the COVID-19 pandemic	
Yes	138 (55.2)
No	112 (44.8)

Analyzing the participant responses in terms of the different e-learning platforms used, the majority reported using either the Blackboard Learning Management System (LMS) (Reston, VA: Blackboard Inc.) (84.8%), web-based meeting applications, such as Zoom (San Jose, CA: Zoom Video Communications Inc.) (82.8%), or Microsoft Teams (Redmond, WA: Microsoft Corporation) (64.4%). The least popular applications in terms of usage were Facebook (Menlo Park, CA: Meta Platforms Inc.), Webex, and GoTo Meeting (Boston, MA: GoTo Inc.) (all less than 10%). A minority of participants also mentioned using Google Classroom (Mountain View, CA: Alphabet Inc.)(33.2%), PowerPoint (Redmond, WA: Microsoft Corporation) presentations with recorded audio (22%), and email-based learning resources (36.8%) as a means of e-learning. Most participants reported that e-learning enabled them to study from home (72.8%), helped them access course content easily (68.4%), provided an opportunity to review recorded course content (64.8%), and offered a comfortable learning environment (58.8%). Very few participants found e-learning advantageous in terms of interactivity (12%) or receiving feedback (18%). In contrast, the absence of technical issues (76.4%) and interactions with the course faculty (45.2%) were perceived as the greatest advantages of classroom learning. Practicing self-discipline (34.8%), minimal distractions (36.4%), scope for social interaction (27.2%), and school-based learning conditions (20.8%) were seen as advantages of classroom learning by only a few participants (Table 2).

**Table 2:** Participant responses towards usage of different e-learning platforms and digital tools and the advantages of e-learning and classroom learning (n=250). ^#^Multiple response datasets. LMS: Learning Management System

Categories of participant responses^#^	n (%)
Different e-learning platforms and digital tools used	
Blackboard LMS	212 (84.8)
Google classrooms	83 (33.2)
Zoom application	207 (82.8)
Microsoft Teams	161 (64.4)
Skype	16 (6.4)
Audio recorded PowerPoint	55 (22.0)
Webex	6 (2.4)
GoTo Meeting	6 (2.4)
Emails	92 (36.8)
Advantages of e-learning	
Ease of access to course content	171 (68.4)
Study at your own pace	117 (46.8)
Ability to study from home	182 (72.8)
Interactivity in class	30 (12.0)
Ability to record and review course content	162 (64.8)
Learning under comfortable surroundings	147 (58.8)
Quick feedback	45 (18.0)
Ease of managing time	90 (36.0)
Advantages of classroom learning	
Greater interaction with course faculty	113 (45.2)
No chance of technical problems	191 (76.4)
Better learning conditions at school	52 (20.8)
Ability to practice self-discipline	87 (34.8)
Better scope for social interaction	68 (27.2)
No or minimal distractions	91 (36.4)

Comparing participants’ responses in favor of either e-learning or classroom learning with respect to different teaching methodologies provided mixed results. While the majority of participants found both methods acceptable for problem-based learning sessions (47.6%) and theoretical lectures (39.3%), they reported e-learning to be less effective than classroom learning for clinical (54.6%) and practical sessions (46.6%). The detailed frequencies of the participants’ responses comparing e-learning and classroom learning for the different teaching methodologies are presented in Table 3.

**Table 3:** Participant responses comparing e-learning and classroom learning for different teaching methodologies ranked on a four-point scale. ^#^Simulated clinical/practical sessions for e-learning.

Ranked responses for different teaching methodologies	n (%)
Problem-based learning (n=233)	
E-learning is as good as classroom learning	48 (20.6)
E-learning is better than classroom learning	54 (23.2)
Both learning methods are acceptable	111 (47.6)
E-learning is poorer than classroom learning	20 (8.6)
Theoretical lectures (n=247)	
E-learning is as good as classroom learning	76 (30.8)
E-learning is better than classroom learning	57 (23.1)
Both learning methods are acceptable	97 (39.3)
E-learning is poorer than classroom learning	17 (6.9)
Clinical sessions^#^ (n=183)	
E-learning is as good as classroom learning	16 (8.7)
E-learning is better than classroom learning	12 (6.6)
Both learning methods are acceptable	55 (30.1)
E-learning is poorer than classroom learning	100 (54.6)
Practical sessions^#^ (n=208)	
E-learning is as good as classroom learning	22 (10.6)
E-learning is better than classroom learning	22 (10.6)
Both learning methods are acceptable	67 (32.2)
E-learning is poorer than classroom learning	97 (46.6)

When participants were asked to respond to e-learning as a preferred method over classroom learning on a five-point nominal scale, most responses were neutral or uncertain. There was a unanimous agreement (agree/strongly agree) recorded in favor of e-learning, primarily because of its ability to cover course objectives and classroom learning (44%) within prescribed time, and also its modern nature (58%), and the lower time and effort associated with using it (60.8%). Conversely, there was general disagreement among participants (strongly disagree/disagree) on e-learning leading to better student-faculty interaction (57.2%), or facilitating student participation, when compared with classroom learning (40.4%) (Table 4).

**Table 4:** Participant responses towards e-learning as a preferred method of learning over classroom learning ranked on a five-point scale (n=250).

Points for preference of e-learning over classroom learning	Classification of ranked responses, n (%)				
	Strongly disagree	Disagree	Neutral/uncertain	Agree	Strongly agree
E-learning is more comfortable than classroom learning	27 (10.8)	70 (28.0)	83 (33.2)	43 (17.2)	27 (10.8)
E-learning methods motivate me as a student (n=230)	18 (7.8)	60 (26.1)	90 (39.1)	60 (27.0)	-
E-learning enables coverage of course objectives as good as in-classroom learning	13 (5.2)	59 (23.6)	68 (27.2)	81 (32.4)	29 (11.6)
E-learning is more modern than classroom learning	23 (9.2)	27 (10.8)	55 (22.0)	98 (39.2)	47 (18.8)
E-learning is more fun than classroom learning	38 (15.2)	50 (20.0)	92 (36.8)	46 (18.4)	24 (9.6)
E-learning provides scope for more lecturers than classroom learning	18 (7.2)	37 (14.8)	92 (36.8)	67 (26.8)	36 (14.4)
E-learning has better student-to-faculty interaction than classroom learning	53 (21.2)	90 (36.0)	64 (25.6)	29 (11.6)	14 (5.6)
Ability to understand course contents is better in e-learning than in classroom learning	31 (12.4)	61 (24.4)	95 (38.0)	40 (16.0)	23 (9.2)
Student participation is easier in e-learning than in classroom learning	43 (17.2)	58 (23.2)	83 (33.2)	42 (16.8)	24 (9.6)
E-learning needs less time and effort than classroom learning	19 (7.6)	22 (8.8)	57 (22.8)	86 (34.4)	66 (26.4)

Comparing the mean ranks of the ordinal responses for the different teaching methodologies of e-learning and classroom learning with the categorical variables, no statistically significant interaction was observed, except for the level of study in theoretical lectures, and the level of IT skills in practical sessions (Table 5). Regarding the level of study, students belonging to the junior levels (third, fourth, and fifth years), rather than interns and post-graduate students, preferred classroom-based lectures. Similarly, students with self-perceived below-average IT skills lacked the confidence to gain knowledge through practical e-learning sessions, even though they were simulated.

**Table 5:** Effect of demographic characteristics and previous digital learning experience on the mean ranks of participant responses towards different teaching methodologies of e-learning and classroom learning. (Higher mean rank score indicates preference for classroom learning). ^#^Simulated clinical/practical sessions for e-learning. COVID-19: coronavirus disease 2019

Demographic characteristics and digital learning experience	Problem-based learning		Theoretical lectures		Clinical sessions^#^		Practical sessions^#^	
	Mean ranks	p-Value	Mean ranks	p-Value	Mean ranks	p-Value	Mean ranks	p-Value
Age of the participants								
<21	110.74	0.87	130.23	0.831	96.88	0.444	99.43	0.065
22-25	117.76		124.08		89.54		100.41	
>26	117.71		120.09		101.9		124	
Gender distribution								
Male	111.84	0.248	119.1	0.281	86.78	0.212	102.62	0.653
Female	121.38		128.41		95.71		106.11	
Level of study in dental school								
Third-year student	110.6	0.088	141.57	0.002	97.53	0.235	103.72	0.55
Fourth-year student	128.51		124.18		95.94		98.78	
Fifth-year student	117.37		142.35		88.27		105.37	
Intern	89.04		92.79		74.1		99.21	
Postgraduate student	120.63		103.09		100.09		118.56	
Level of information technology skills								
Above average	124.81	0.356	118.44	0.785	87.58	0.239	92.69	0.03
Average	117.49		125.82		90.37		102.8	
Below average	102.13		120.35		107.89		131.78	
Prior e-learning experience before the COVID-19 pandemic								
Yes	122.49	0.13	126.71	0.479	89.26	0.387	102.25	0.543
No	109.82		120.57		95.38		106.97	

When the mean ranks of the nominal responses for e-learning as a preferred method over classroom learning were compared with the categorical variables, no statistically significant interactions were observed for demographic characteristics, IT skill levels, or prior experience with e-learning (Table 6).

**Table 6:** Effect of demographic characteristics and previous digital learning experience on the mean ranks of participant responses towards e-learning as a preferred method of learning over classroom learning. A higher mean rank score indicates preference for e-learning. COVID-19: coronavirus disease 2019

Demographic characteristics and digital learning experience	Mean (SD)	t-value/F-value	p-Value
Age of the participants			
<21	30.32 (4.0)	1.566	0.211
22-25	29.63 (5.9)		
>26	31.50 (7.2)		
Gender distribution			
Male	29.68 (6.4)	-0.79	0.43
Female	30.32 (5.7)		
Level of study in dental school			
Third-year student	29.23 (6.6)	1.641	0.165
Fourth-year student	30.26 (5.1)		
Fifth-year student	30.87 (6.1)		
Intern	27.79 (5.6)		
Postgraduate student	30.88 (7.1)		
Level of information technology skills			
Above average	29.0 (7.5)	1.869	0.157
Average	30.46 (5.3)		
Below average	28.33 (8.4)		
Prior e-learning experience before the COVID-19 pandemic			
Yes	30.30 (6.4)	0.769	0.443
No	29.68 (5.7)		

## Discussion

Effectiveness of educational study models is often assessed using three primary characteristics as follows: student study experiences, synchronous student participation, and faculty-student interaction [16]. In any interactive learning process, students develop knowledge, skills, and new research ideas through active and synchronized participation in the course, and interaction is facilitated by the faculty members [17]. Traditional classroom learning models are face-to-face and didactic in nature, wherein the prescribed study material is handed over to students through lectures, faculty-student discussions, and study handouts [10,18]. On the other hand, e-learning is relatively asynchronous, because of a physical gap in communication between faculty and students, thereby leading to remote communication of course content and study materials [7]. Nevertheless, with the advancement in technology relating to internet-based audio-visual aids, such as chat, virtual classrooms, and online meeting platforms, e-learning has evolved to be more synchronous than before [7]. In light of the extensive use of e-learning during the recent COVID-19 pandemic-induced lockdowns and mandated closures of dental and medical schools, it is alluring to ask the question if electronic learning will predominate over classroom learning in the near future. Therefore, the present study evaluated student perceptions regarding e-learning in comparison to classic classroom learning, with a focus on dental education at public universities in Riyadh, Saudi Arabia.

Integration of the dental curriculum with e-learning was a welcome sign during the COVID-19 pandemic. However, the advantages and disadvantages of the replacement of traditional classroom learning with e-learning, especially in the context of dental education, require thorough analysis. The dental curriculum in Saudi Arabia extends over a period of five years and includes exclusive didactic learning methodologies in the first and second preclinical years, followed by three years of combined clinical and didactic training. In addition, dental students undergo training for psychomotor skills through the simulation of laboratory phantom models [8]. Over the past few years, several dental institutions in Saudi Arabia have introduced online platforms, such as Blackboard LMS, which were used mainly for theoretical interactions and delivering written assessments [8]. However, this always involved a combined strategy, wherein face-to-face training was always part of the learning methodology [19]. It was reported as early as 2008, that adopting e-learning as a standalone teaching platform for medical or dental education should only be considered after evaluating the benefits to the student community and its degree of adaptability at large [20]. However, a paradigm shift towards e-learning as a prime teaching modality of the dental curriculum occurred due to circumstances surrounding the COVID-19 pandemic [11,13].

In this study, the ability of e-learning to provide learners with materials that are accessible at any convenient time for self-instruction and collaborative dental education was reported as advantageous. Nevertheless, while this type of asynchronous learning is more beneficial for students in their early dental school years, it could pose challenges for students undergoing practical or clinical courses, and postgraduate fellows and residents with clinical rotations. Especially considering the fact that almost all clinical courses, either at the undergraduate or at the postgraduate levels, are associated with limited hours of hands-on training and mandatory curriculum requirements [21]. The complexity of the dental curriculum and the lack of technical expertise among faculty members to provide simulated practical and dental clinical training have restricted e-learning. The current study observed that most students in the clinical year were disappointed with this mode, expressing difficulties in coping with practical and clinical sessions. In the future, the orientation of faculty and administrators in system deployment, content development, and designing learning pathways and programs is vital to support the replacement of classical face-to-face learning by e-learning [22]. Interestingly, the evolution of virtual patient simulations and high-impact e-learning materials has encouraged educators and learners to adopt this new learning platform [23].

E-learning platforms currently focus on individualized (adaptive) and collaborative learning, and they transform educators from disseminators to facilitators [24]. The present study sample appreciated e-learning as being advantageous in terms of personalized content delivery, identification of the students' potential, and constant monitoring of their progress [25]. Advanced communication platforms have enabled more interactive and collaborative e-learning experiences [15]. The study participants found noticeable improvements in the level of knowledge, assimilation of current concepts, fulfillment of designated course objectives, and students' satisfaction with e-learning, which may lead to transformations, even in the prevailing clinical protocols [26]. However, the paradigm shift to the new platform is questionable because of the continuous learning required in dental education, along with clinical and practical competency development to adapt to evolving technologies in dental management [27]. It is in this context that clinical learning is best achieved through face-to-face interactions, as it helps students gain professionalism about the compassionate expressions and body language that should be adopted during patient handling [28]. This has been reiterated in an earlier study, wherein medical students get trained in perceiving visual information relating to subtle emotions only through face-to-face interaction and never from reading texts or attending lectures [29]. The same sentiment was echoed with respect to clinical training in the present study findings too.

In recent decades, e-learning has gained importance in dental education, with students becoming more acquainted with the internet and gadgets, such as computers and smartphones [6]. Garnering essential IT skills has become an imperative part of not only learning activities but also for day-to-day lifestyle and sustenance. In the present study, self-reported IT skills based on an individual’s own assessment were carried out to provide their personal judgment and feedback as to how well they are capable of handling IT-based interactions. The sample group demonstrated a high level of command in IT skills and used Blackboard, Zoom application, Microsoft Teams, and Google Rooms, with relative ease and confidence. However, even today, most students in master’s programs use e-learning platforms only in literature searches for thesis completion [30]. Hence, compared to other fields of education, dental students are still at a nascent stage with respect to e-learning [31]. Current statistics indicate that academicians have increased in number, as the dental fraternity becomes increasingly popular [31]. Therefore, continuous updates are mandatory for the faculty handling e-learning platforms [32]. The quality of dental education and student satisfaction is dependent on proper design, teaching, and tutoring in updated e-learning platforms [33]. Traditional classroom learning possesses the advantage of face-to-face interaction and tutor support, which e-learning platforms do not offer [32,34]. Therefore, although problem-based learning (PBL) and theoretical sessions did not differ much between the two modes of teaching, accommodating practical and clinical sessions in e-learning was challenging. However, with advances in technology, faculty and students should accommodate these changes and update their IT skills [22,33]. For transformation into an e-learning platform for dental education, students’ comfort and motivation in grasping IT concepts should be assessed to avoid skepticism [35]. Furthermore, since e-learning is more prevalent in English, language barriers may hinder its implementation as the primary teaching modality [6]. Educational institutions should provide academicians with the resources, adequate equipment, and necessary manpower to successfully incorporate e-learning into the present system [3].

Whether we prefer it or not, digital trends are bound to be the norm rather than the exception in the near future. Especially with the advent of generative artificial intelligence (AI), learning at a self-styled pace with the help of AI chatbots and applications is no longer a utopian reality [36]. In light of these developments, it is imperative that e-learning is blended into mainstream traditional learning for all streams of education and curricula. Although it is easier to incorporate e-learning for self-study and lecture-based learning, it should be taken further toward cognitive learning through problem-solving and scenario-based learning methodologies. This has already been experimented with and implemented for medical education, which does not require training in psychomotor skills [7,22]. As for practical and clinical training of psychomotor abilities, such as that required in the dental curriculum, further research and development need to be directed towards the development of the same.

## Conclusions

This research revealed the preferences of dental students through a detailed comparison of the traditional classroom and e-learning modalities, including an assessment of various aspects of PBL, practical and clinical sessions. While e-learning enables enhanced performance and learning capacity, the advantages of personal interactions and the feasibility of practical and clinical sessions are achieved only through the classroom model. Periodic evaluation of students’ satisfaction and their effective coping mechanisms with newer teaching methods will help prioritize learners’ interests and enable comprehensive improvement in the education system.
